# Emerging Roles of lncRNAs in the Formation and Progression of Colorectal Cancer

**DOI:** 10.3389/fonc.2019.01542

**Published:** 2020-01-17

**Authors:** Qinglian He, Jiali Long, Yuting Yin, Yuling Li, Xue Lei, Ziqi Li, Wei Zhu

**Affiliations:** ^1^Department of Pathology, Guangdong Medical University, Dongguan, China; ^2^Department of Pathology, Dongguan Hospital of Southern Medical University, Dongguan, China

**Keywords:** lncRNAs, colorectal cancer, transcription, polypeptide, treatment

## Abstract

Colorectal cancer (CRC) is the primary cause of cancer-related death worldwide; however, specific and sensitive tools for the early diagnosis and targeted therapy of CRC are currently lacking. High-throughput sequencing technology revealed that gene expression of long-chain non-coding RNAs (lncRNAs) in a number of cancers directly or indirectly interferes with various biological processes. Emerging evidence suggests that lncRNAs regulate target genes and play an important role in the biological processes of malignancies, including CRC. Many carcinostatic/oncogenic lncRNAs have been identified as biomarkers for metastasis and prognosis in CRC; hence, they serve as therapeutic tools. In this article, we systematically review the literature on the disordered lncRNAs in CRC from four aspects: DNA transcription, RNA level regulation, post-translational level, and the translation of lncRNAs into polypeptides. Subsequently, we analyze the mechanism through which lncRNAs participate in the biological process of CRC. Finally, we discuss the application and prospects of these lncRNAs in CRC.

## Introduction

Long-chain non-coding RNA (lncRNA) is a transcript with a length >200 nucleotides. The estimated number of lncRNA genes in the human genome is 16,000 (1). According to the relative position of the lncRNA and protein gene in the genome, lncRNAs are identified into five types: sense lncRNAs, antisense lncRNAs, bidirectional lncRNAs, intronic lncRNAs, and intergenic lncRNAs (also termed long intergenic non-coding RNAs [lincRNAs]) (2). Recently, lncRNAs have been demonstrated to exert a crucial effect on a variety of biological activities by affecting gene expression in different types of cancer (3, 4). Furthermore, abnormal expression of lncRNAs is associated with invasion, metastasis, chemoresistance, and resistance to radiation in CRC (5, 6). For example, lncRNA H19 modulates the expression and transcription of cyclin genes, facilitates the levels of epithelial-mesenchymal transition (EMT)-related genes, and plays an essential role in metastasis, apoptosis, autophagy, and signal transition in CRC (7–9). The upregulation of lncRNA H19 is deemed to be a key prognostic element for patients with CRC (7). In this article, we highlight the role of lncRNAs in CRC and the biological processes involved in CRC. In addition, we emphasize that lncRNAs are highly promising biomarkers for CRC and are expected to be used in the clinical setting.

## Mechanisms of lncRNAs in CRC

**Figure 1:**
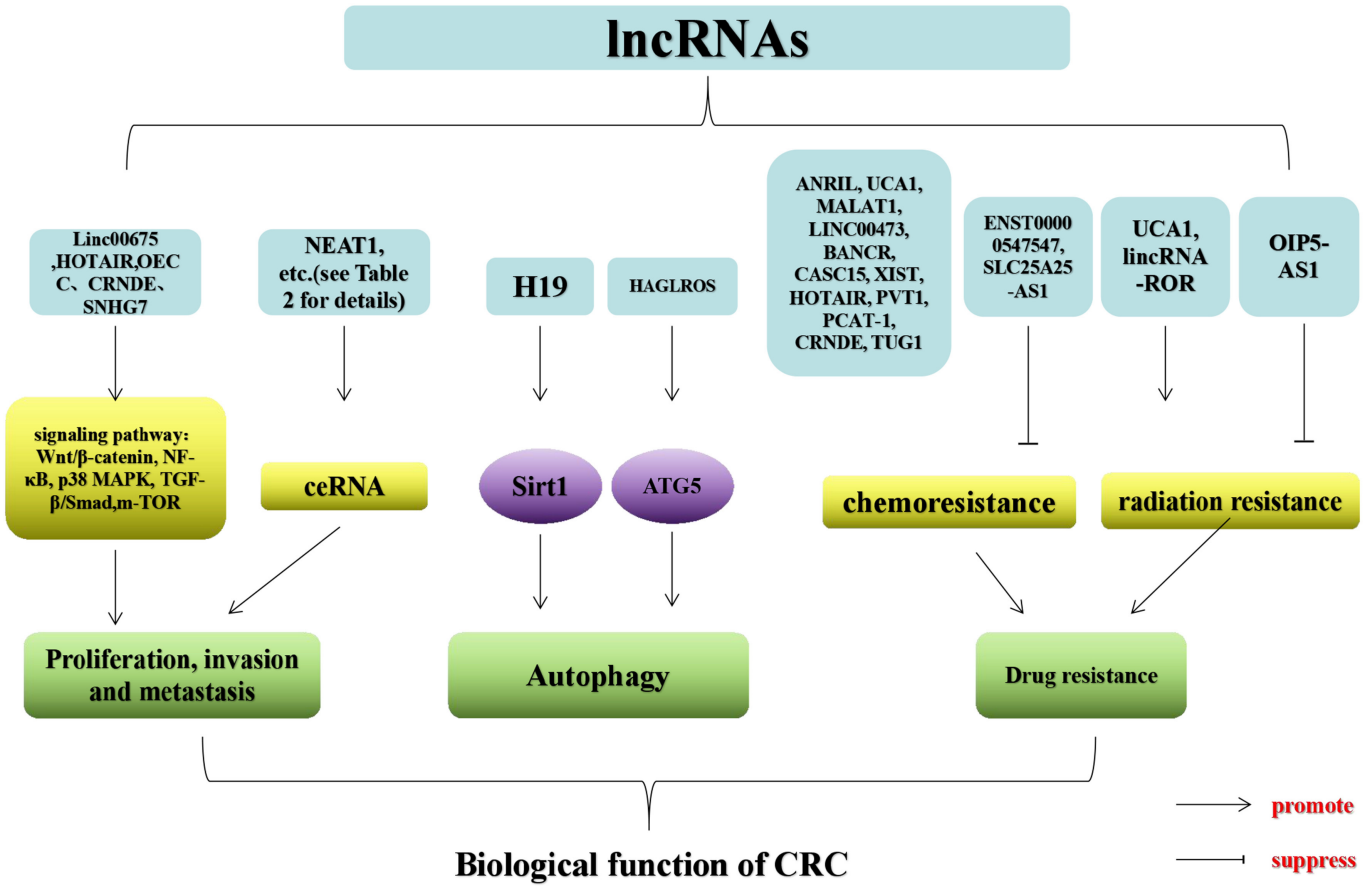
Regulatory mechanisms of lncRNAs that may be associated with CRC. SWI/SNF (chromatin remodeling complex); BAF170 (SWI/SNF-related, matrix-associated, actin-dependent regulator of 67 chromatin subfamily c member 2); lncRNA TCF7 (lncRNA T-cell factor 7); TFs (transcription factors); RISC (RNA-induced silencing complexes); SF (splicing factor); hnRNP1 (heterogeneous nuclear ribonucleoprotein 1).

lncRNAs can modulate gene expression in assorted aspects (e.g., chromosomal remodeling, transcription, and post-transcriptional processing) (10, 11). In this article, we elaborate on the mechanism through which lncRNAs act on gene expression in CRC in four aspects (DNA transcription, RNA level regulation, post-translational level, and self-translation of lncRNAs into polypeptides), which in turn affect the biological behavior of CRC (Figure 1).

### lncRNAs and DNA Transcription

It is well-established that the DNA transcription process is mainly divided into two steps. The first step is the synthesis of the original transcription products (including the initiation, extension, and termination of transcription). The second step is the post-processing of these transcription products to transform them from their inactive form into mature RNA with biological functions. This part mainly reviews the functions of lncRNAs in the modulation of the first step of DNA transcription. The second step of DNA transcription at the RNA level will be discussed in the succeeding part.

Depending on the subcellular position and origin of lncRNAs, they can be divided into nuclear lncRNAs and mitochondrial lncRNAs (12). Numerous lncRNAs reside in the nucleus, concentrated in particular subnuclear chambers, or abundant in chromatin (12). It was described that nuclear lncRNA is associated and involved in several biological processes that affect DNA transcription (e.g., chromatin organization, transcriptional and post-transcriptional levels, and serve as structural scaffolds for nuclear domains) (1). Numerous nuclear lncRNAs are associated with chromatin; nuclear lncRNAs are classified into three basic types: chromatin-rich RNAs, chromatin-associated lncRNAs, and GAA repeat-containing RNAs (12). Some nuclear lncRNAs contain both DNA- and protein-binding motifs, which can carry polycomb repressive complexes lacking sequence-specific DNA binding motifs to specific genomic loci, thus altering the transcriptional activity of genes on chromosomes. For example, in hepatoma cells, transcription of the T-cell factor 7 (TCF7) gene is facilitated by lncRNA TCF7 via the recruitment of SWI/SNF complexes with the lncRNA TCF7 promoter (13). Likewise, recent research manifested that lncRNA TCF7 recruits BAF170 (SWI/SNF-related, matrix-associated, actin-dependent regulator of chromatin subfamily c member 2) to stimulate the TCF7 promoter and alter the expression of TCF7 in CRC (14). The data indicated that overexpression of lncRNA TCF7 can promote the occurrence of malignant behavior of CRC cells transfected with small interfering-lncRNA TCF7, thereby reversing the effect of lncRNA TCF7 on migration and invasion of CRC cells (14). The interplay between lncRNAs and DNA-binding proteins prevents these proteins from entering the DNA recognition element, thus inducing or inhibiting transcription dependent upon the nature of the target protein (15). In this regard, it becomes clear that lncRNA growth arrest-specific transcript 5 (GAS5) can cooperate with the DNA binding site of the glucocorticoid receptor and compete with the glucocorticoid response element on DNA. This prevents the glucocorticoid receptor from entering the target DNA (16). Based on quantitative reverse transcription polymerase chain reaction (qRT-PCR), lncRNA GAS5 was shown to be downregulated in CRC (17). The expression of lncRNA GAS5 correlates with the degree of differentiation, metastatic capacity, and stage of lymph node metastasis (17). Moreover, the reduced expression of lncRNA GAS5 accelerates cellular proliferation and angiogenesis, influences the cell cycle and apoptosis, and is a prognostic biomarker of CRC (18, 19). Nevertheless, in a recent observation, it was demonstrated that lncRNA GAS5 conduces to lymphatic metastasis of CRC (19). Additionally, using qRT-PCR, Liu et al. detected upregulation of lncRNA GAS5 and downregulation of miR-221 in CRC (20). Surprisingly, the level of lncRNA GAS5 is also related to clinical and pathological features (20). Therefore, the mechanism of lncRNA GAS5 in CRC warrants further study and confirmation.

Similarly, lncRNA P21-associated non-coding RNA DNA damage-activated (PANDA) interacts with the transcription factor nuclear factor Y-box A to reduce the occupancy of nuclear factor Y-box A at the chromatin-containing target gene (21). Studies have found that the expression of lncRNA PANDA is a predictor of prognosis in patients with CRC. Moreover, lncRNA PANDA is a vital risk factor affecting the lifetime of these patients (22). Furthermore, the results of a multivariate analysis implied that the expression of lncRNA PANDA is a factor that can directly affect the prognosis of CRC, while *in vitro* studies suggested that lncRNA PANDA may promote CRC transfer via the EMT pathway (23). lncRNAs activates or inhibits transcription by a local action (near its transcriptional site [cis-regulation]) or a distal action (a site located on another chromosome [trans-regulation]). lncRNAs regulate transcription by affecting transcription factor (TF) activity. A published study identified the function of nuclear lncRNAs in modulating the level of the tumor suppressor protein p53 (24). In CRC, lncRNA p53 upregulated regulator of p53 levels (PURPL) blocks the assembly of the Myb-binding protein 1A (MYBBP1A)-p53 complex by binding to the p53-stabilizing protein MYBBP1A, thereby weakening the cell bank of p53 (24). In this instance, lncRNA PURPL indirectly regulates transcription by modulating the expression of principal TF, p53 (24). lncRNAs can participate in direct transcription by cooperating with transcriptional complexes or DNA components. RNA-binding proteins (RBPs) can cooperate with single- or double-stranded RNA and affect the post-transcriptional modulation of corresponding gene expression (25). Notably, some RBPs can be used as transcriptional regulators, while lncRNAs can regulate their activity. LncRNA colon carcinoma-1 (OCC-1) regulates large amounts of mRNA at the post-transcriptional level by affecting the stability of RBP human antigen R (26). Consequently, lncRNAs may be involved in DNA transcription in CRC through an interplay with chromatin regulatory proteins, DNA-binding proteins, and cis or trans elements.

### RNA Level Regulation

LncRNA is a non-negligible element in the regulation of the progression of CRC at the RNA level, mainly composed of mRNA and microRNA (miRNA). Regarding the regulation of miRNAs by lncRNA, some scholars concluded four ways in which lncRNAs interact with miRNAs, namely host genes as miRNAs, miRNA instability, mRNAs that compete with miRNAs, and capture of miRNAs (27). Firstly, lncRNAs can serve as a precursor sequence of miRNAs. By existing data analysis, some lncRNAs have been discovered to act as host genes of miRNAs (28), such as lncRNA plasmacytoma variant translocation 1 (PVT1) and lncRNA AK058003 (29). Wang et al. used the Gene Expression Omnibus database, clinical sample measurements, and further multivariate analysis to conclude that the expression of lncRNA PVT1 is negatively correlated with prognosis and the increased level of lncRNA PVT1 is the main factor triggering CRC progression (30). Moreover, knockout of lncRNA PVT1 in CRC cells resulted in inhibition of invasion, migration, and proliferation. Fan et al. discovered that knocking out lncRNA PVT1 may reverse multidrug resistance in CRC cells (31). Secondly, miRNAs have a negative impact on the stability of lncRNA. For instance, the let-7 family binds to the RBP HuR (32), which reduces the stability of lncRNA p21 (33). The level of lncRNA p21 is reduced in CRC cells and tissue samples (34, 35). A study showed that miR-451 regulates lncRNA p21 delivery, thereby inhibiting β-catenin signaling and the oncogenicity of CRC stem cells (36). Thirdly, lncRNAs are considered to regulate the expression of miRNAs through linking with miRNAs or their target. LncRNA beta-secretase 1 antisense RNA (BACE1AS) can inhibit the target of miR-485-5p BACE1 by miR-485-5p, thereby alleviating the inhibition of BACE1 (37). The last and common way is that lncRNA, which is a competitive endogenous RNA (ceRNA), plays a sponge role in competitively binding miRNAs to inhibit their binding activity. Through high-throughput sequencing technology, a number of studies investigating lncRNA as ceRNA have been performed (38, 39). This article summarizes the latest research on lncRNA as a ceRNA in CRC (Supplementary Table 1).

In the aspect of mRNA, lncRNA regulation is mainly involved in the modification of its stability and splicing. It is established that the lifespan of mRNAs is considerably short, especially for the oncogenes, such as cyclin D1 and c-Myc (40, 41). Thus, once their mRNA stability is increased, it may give rise to oncogenesis. In CRC cells, lncRNA Assisted Stabilization of Transcripts (LAST) can interact with the CCHC-type zinc finger nucleic acid-binding protein to modulate the stability of cyclin D1 mRNA (42). Furthermore, the stability of c-myc is selectively regulated by lincRNA-regulator of reprogramming (lincRNA-ROR) (43). Researchers found that lincRNA-ROR is upregulated in CRC cells and tissues, and further investigations manifested that knockout of lincRNA-ROR advocates sensitivity to radiation therapy against CRC (44). Alternative splicing refers to the process in which eukaryotic cells (including cancer cells) selectively splice different splice sites of precursor mRNA to form various mature mRNAs. These mRNAs are subsequently translated into multiple proteins with diverse biological functions (45). Hence, alternative splicing can affect the origination and progression of cancer (46). Moreover, many cis-acting elements and trans-acting factors are involved in the regulation of splicing (47). The cis-acting elements of RNA that participate in selective splicing include enhancers and inhibitors. Heterogeneous nuclear ribonucleoprotein (hnRNP) and serine/arginine-rich protein (SR protein) are trans-acting factors (45). Thus, regulation of gene splicing is frequently intricate. At present, an increasing body of evidence indicates that lncRNAs may be a key factor in selective splicing (48). The interaction of lncRNAs with hnRNPs may enhance their combination with exon-splicing silencers or intron-splicing silencer elements in mRNA alternative splicing (49–51). For instance, lncRNA metastasis-associated lung adenocarcinoma transcript 1 (MALAT1) can modulate alternative splicing of all types of genes by linking to serine-arginine (SR) proteins, as well as affecting their corresponding subnuclear localization (52). In solid tumor cells, upregulation or depletion of lncRNA MALAT1 alters the splicing of pre-mRNA of several SR protein splicing factor 1 (SRSF1) target genes (53, 54). In hepatic carcinoma, knockdown of SRSF1 abolishes the carcinogenic characteristic of cells upregulating lncRNA MALAT1 (55). In CRC, lncRNA MALAT1 can bind to splicing factor proline and glutamine rich (SFPQ) (also termed PSF [PTB-associated splicing factor]) and enhance the release of polypyrimidine tract cooperating with protein 2 (PTBP2) from the SFPQ/PTBP2 complex (56). A recent report indicated that lncRNAs are associated with RNA precursor processing (56). The lncRNA cancer susceptibility 9 (CASC9) and hnRNP complexes affect AKT signaling and DNA damage in HCC (57). HnRNPL is one of the proteins that stably bind to the hnRNP complex, and together with other hnRNP proteins, may exert a major effect on the formation, packaging, processing, and function of RNA.

### Post-translational Level

Mammalian lncRNAs regulate transcription; however, their effects at the post-translational level is currently under investigation. Numerous data have emerged indicating that lncRNAs can influence gene epigenetics by recruiting chromatin-modifying enzymes. For example, lncRNA HOX transcript antisense intergenic RNA (Hotair) can be combined with polycomb repressive complex 2, affecting cancer metastasis (58). Under these circumstances, histone H3 lysine 27 (H3K27) methylated state and gene expression are changed to advance neoplastic invasion by lncRNA Hotair. Additionally, lncRNA Hotair can be used as a scaffold to select histone-modifying enzymes and affect the expression of specific genomes (59). Therefore, inhibition of lncRNA Hotair leads to a decrease in cellular invasion (59). LncRNA Hotair was found in breast cancer as a support for Hepatitis B X-interacting protein and lysine demethylase 1, and is subsequently mediated by c-Myc (60). When the level of human antigen R in cellular processes (e.g., cell senescence) declines, lncRNA Hotair also plays a scaffolding role in protein ubiquitination (61). LncRNA Hotair forms a complex with two E3 ubiquitin ligases Dzip3 (DAZ-interacting zinc finger protein 3) and Mex3b (mex-3 RNA-binding family member B) carrying an RNA binding domain. Moreover, lncRNA Hotair also cooperates with respective ubiquitinated substrates, Snurportin-1, and Ataxin-1 (61). Through advancing the emergence of the complex, the ubiquitinated Ataxin-1 and Snurportin-1 are advocated by lncRNA Hotair to enhance their degradation. Furthermore, the level of lncRNA Hotair is upregulated in CRC (62). Studies have shown that knockdown of lncRNA Hotair markedly inhibits cellular proliferation and the formation of clones, indicating that CRC neoplasia could be accelerated by lncRNA Hotair (62, 63). EZH2 (enhancer of zeste homolog 2), a histone methylase, an epigenetic modification regulator, is up-regulated in CRC (64). Also, knockout of EZH2 considerably inhibits colony formation and cell viability. In addition, RNA immunoprecipitation assays showed that lncRNA Hotair combines directly with EZH2 in CRC (64). The lncRNA Angelman (ANCR) syndrome chromosome region, which is detected to be downregulated in CRC tissues and cells, can also specifically bind to EZH2 to suppress progression (65). In breast cancer, lncRNA ANCR syndrome chromosome region regulates the stability of EZH2. It can promote ubiquitination of EZH2, thus accelerating the degradation primarily by enhancing the mutual effect of cyclin-dependent kinase 1 and EZH2. This process increases the phosphorylation intensity of the two specific threonine sites of EZH2 (66).

### Self-Translation of lncRNAs Into Polypeptides

Previously, lncRNAs were considered non-protein-encoded transcripts; however, recent reports confirmed that lncRNAs can also be translated into peptides, involved in cell metabolism and tumor growth (67, 68). For instance, LINC00961 (a conserved lncRNA) produces a polypeptide called “small regulatory polypeptide of amino acid response” that inhibits the activity of amino acid-mediated mechanistic target of rapamycin 1 (mTORC1) by lysosomes, thereby regulating post-injury skeletal muscle regeneration (67). mTORC1 is involved in cellular protein translation, metabolism, and growth (69, 70). It facilitates cell growth (including tumor cells) by occluding destructive metabolic pathways, such as autophagy (71), which functions as the principal degradation pathway in eukaryocytes (72, 73). Likewise, mTORC1 has been identified as the key factor of EMT, metastasis, and motility of CRC (74). Receptor tyrosine kinase phosphorylation is involved in the regulation of mTORC1-independent autophagy through mTORC2 signaling of CRC (75). A study revealed a circularly encoded 87-amino acid peptide of the lincRNA p53 induced non-coding transcript (PINT) induced by the long intergenic non-protein-encoding RNA p53, which counteracts the proliferation of glioblastoma cells (68). These studies suggest that the self-translation of lncRNAs into polypeptides may exert a crucial influence on the evolvement of CRC.

## Biological Function of lncRNAs in CRC

**Figure 2:**
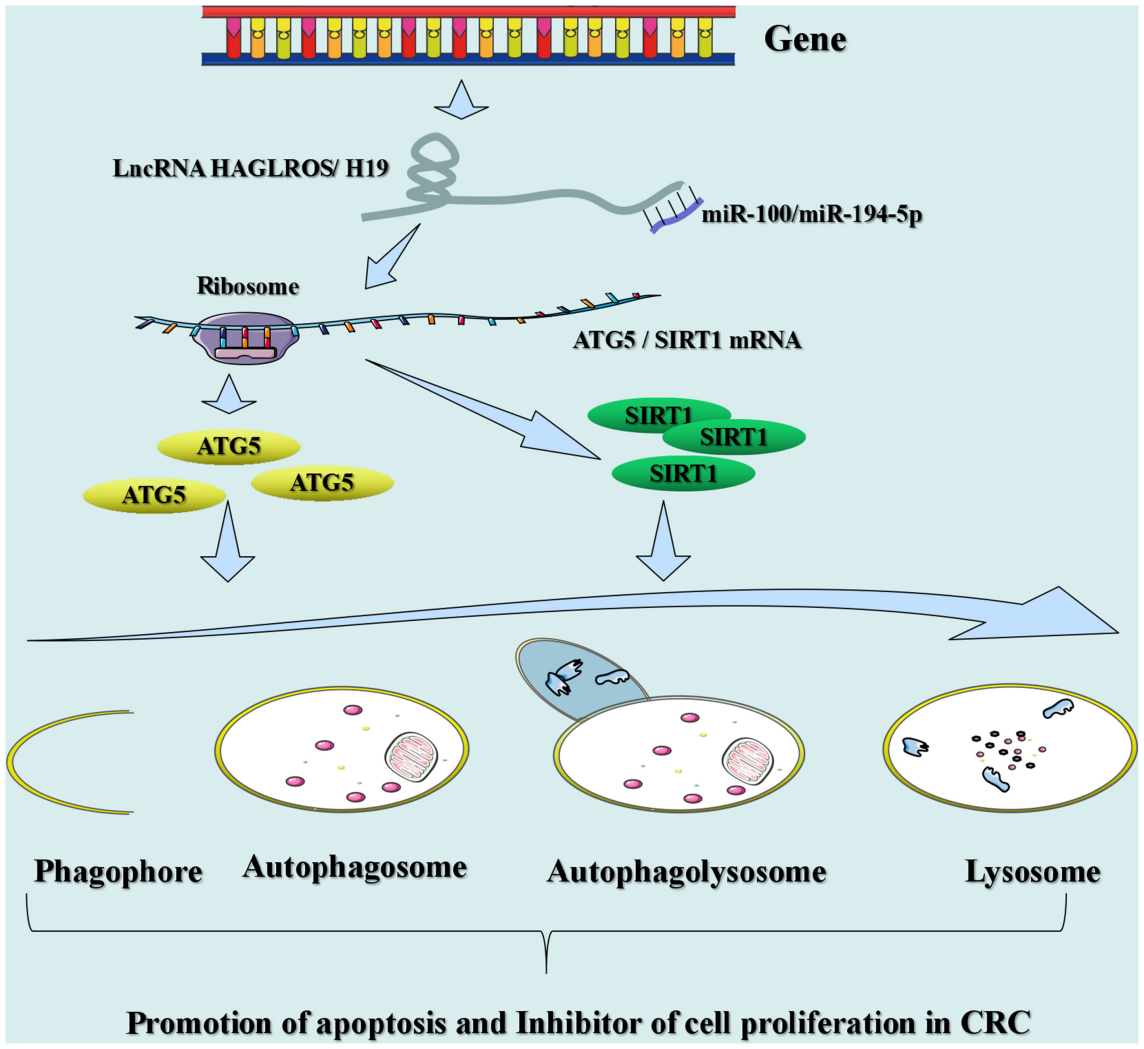
Summary of lncRNAs involved in the biological function of CRC.

Numerous investigations have suggested that lncRNAs engage with several cellular processes, which mainly affect proliferation, invasion, metastasis, autophagy, and drug resistance in CRC. lncRNAs in CRC exert both tumor suppressive and pro-cancer effects, which can be used as novel biomarkers for therapeutic targeting and diagnosis (Figure 2).

### Proliferation, Invasion, and Metastasis

Substantial evidence implies that lncRNAs participate in the proliferation, invasion, and metastasis of CRC. The lncRNAs that exert a promoting effect include lncRNA lung cancer associated transcript 1 (LUCAT1), lncRNA small nucleolar RNA host gene 6 (SNHG6), LINC00483, FAL1 (ATP-dependent RNA helicase), and lncRNA urothelial cancer associated 1 (UCA1) (76–80). The lncRNAs that play an inhibitory role comprise lncRNA runt-related transcription factor 1-intronic transcript 1 (RUNX1-IT1), lncRNA tumor protein p73 antisense RNA 1 (TP73AS1), ENST00000547547, lncRNA heart and neural crest derivatives expressed 2-antisense RNA 1 (HAND2-AS1), etc. (81–84). Some lncRNAs act as ceRNAs to affect proliferation, invasion, and metastasis in CRC (77). Some lncRNAs influence the proliferation, invasion, and metastasis of CRC through Wnt/β-catenin (63, 85), nuclear factor-κB (86), p38 mitogen-activated protein kinase (86), transforming growth factor-β/Smad (87), and other classical signaling pathways (88). Both signaling pathways and ceRNAs affect these processes in CRC. In Supplementary Table 2, we summarized the major tumor suppressor and carcinogenic lncRNAs in CRC.

### Autophagy

**Figure 3:**
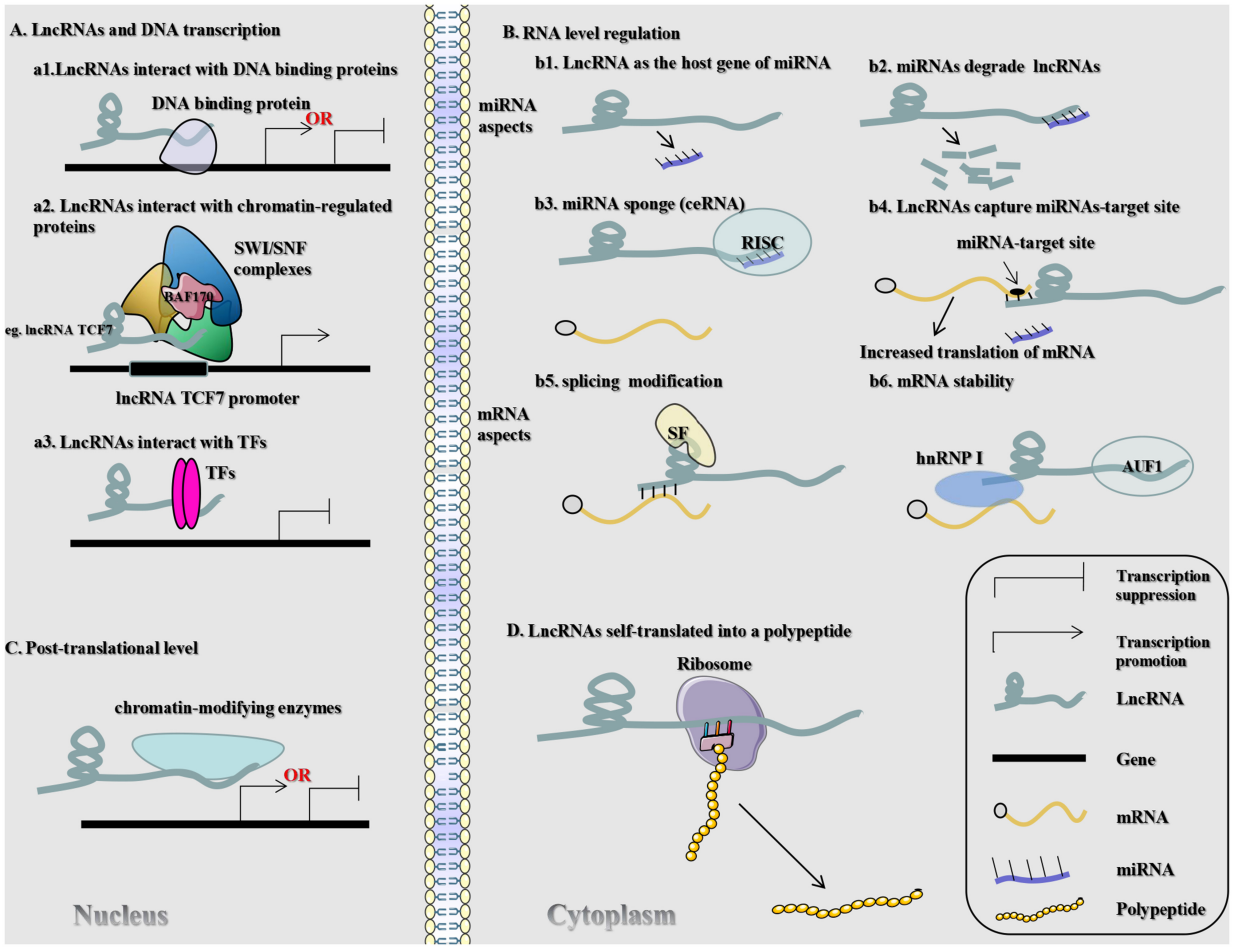
Signaling mechanisms of lncRNAs engaged in autophagy in CRC. LncRNA HAGLROS (H19) as ceRNA sponges miR-100 (miR-194-5p) to repress the degradation of ATG5 (SIRT1) mRNA by miR-100 (miR-194-5p), thereby accelerating autophagy and inhibiting the proliferation of CRC cells.

Autophagy is a cellular process in which cell contents (Cargo) are transported to lysosomes and degraded. This process normally removes proteins, organs, and microorganisms that are abnormal in intracellular function (89). In addition, it plays a critical role in maintaining the homeostasis of cells, tissues, and organs (89). Disorders in autophagy can trigger a range of diseases, including neurodegenerative diseases, inflammation, and cancer (e.g., CRC) (73, 90). Many lncRNAs have been shown to affect autophagy in tumor cells in CRC (Figure 3). For instance, lncRNA HAGLROS (HOXD antisense growth-associated long non-coding RNA opposite strand lncRNA) exerts a sponge function in miR-100 to target the expression of autophagy-related 5 (ATG5) for the regulation of autophagy in CRC cells (91). Knockout of lncRNA UCA1 inhibits cell proliferation in CRC and promotes apoptosis by modulating autophagy (92). LncRNA carbamoyl-phosphate synthase 1-intronic transcript 1 (CPS1-IT1) inhibits the progression and metastatic ability of CRC by impeding autophagy via induction of hypoxia (93). LncRNA H19 may be used as a ceRNA for the sponge miR-194-5p, and studies have found that miR-194-5p directly target sirtuin 1 in CRC cells (9). The final results indicated that lncRNA H19 may affect resistance to 5-fluorouracil (5-FU) in CRC through autophagy (9). Shan et al. found that knockout of the lincRNA-POU domain, class 3, transcription factor 3 (POU3F3) gene enhances autophagy in CRC cells (94).

### Drug Resistance

Currently, chemotherapy and radiotherapy after surgical resection are the most widely utilized treatment strategies for CRC (95, 96). However, chemoresistance and resistance to radiation remain major obstacles to the outcome of patients with CRC. To date, research investigating lncRNAs has yielded some results related to chemoresistance in CRC. For instance, lncRNA antisense non-coding RNA in the INK4 locus (ANRIL) promotes chemoresistance in CRC by modulating the expression of Let-7a and interfering with the expression of ATP-binding cassette subfamily C member 1 (ABCC1) (97). In CRC cells resistant to cetuximab and their exosomes, the expression of lncRNA UCA1 is noticeably upregulated (98). However, the influence of UCA1 on the cetuximab-resistant CRC remains unclear (98). The significant increase in lncRNA MALAT1 is revealed in cells resistant to oxymatrine. However, to a certain extent, silencing of lncRNA MALAT1 can reverse the EMT in cells resistant to HT29 (99). Inhibition of LINC00473 reduced the resistance of CRC cells induced by Taxol *in vitro* and restored the expression of tumor suppressor miR-15a and chemotherapy-induced tumor regression *in vivo*. These findings suggested that LINC00473 may play a role in CRC via miR-15a (100). Knockdown of lncRNA BRAF-activated non-coding RNA (BANCR) may inhibit the progression of CRC and stimulate the sensitivity of CRC cells to adriamycin by acting on the miR-203/chromosome segregation 1-like axis (101). In addition to the development of resistance to the aforementioned chemotherapeutic drugs, some researches have investigated the resistance of CRC to other chemotherapeutic drugs, such as oxaliplatin, doxorubicin (DOX), cisplatin, 5-FU, and methotrexate (MTX). In tissues and cells of oxaliplatin-resistant CRC, lncRNA cancer susceptibility 15 (CASC15) is overexpressed, whereas its knockdown restores the sensitivity of HT29 and HCT116 to oxaliplatin (102). Knockout of lncRNA X inactive-specific transcript (XIST) inhibits resistance to DOX in CRC by up-regulating miR-124 and downregulating serum and glucocorticoid-inducible kinase 1 (103). The level of XIST is markedly upregulated in CRC of DOX resistance, while the level of miR-124 is reversed (103). In CRC cell lines, knockout of lncRNA Hotair and upregulation of miR-203a-3p lead to restrain of cellular proliferation and reduce resistance to cisplatin (63). Notable, knockdown of lncRNA PVT1 suppresses tumor formation and resistance to cisplatin in CRC (104).

At present, the available literature has indicated that lncRNAs promote the efficacy of chemotherapeutic drugs against CRC. Nevertheless, lncRNAs have been found to exert both promoting and inhibitory effects in 5-FU-resistant CRC. LncRNA H19 modulates resistance of CRC to 5-FU by autophagy through sirtuin 1 (9). The reduced level of lncRNA prostate cancer-associated ncRNA transcript 1 (PCAT-1) in CRC cells inhibits motility, reduces their invasive capacity, and increases sensitivity to 5-FU (105). LncRNA Hotair contributes to the development of resistance to 5-FU by inhibiting miR-218 and activating the nuclear factor-κB/thymidylate synthase signaling pathway in CRC (106). Likewise, lncRNA colorectal neoplasia differentially expressed (CRNDE) enhances proliferation and resistance to 5-FU, as a result of Wnt/β-catenin signaling mediated through miR-181a-5p in CRC (85). On the contrary, lncRNA ENST00000547547 (a 434 bp lncRNA on human chromosome12q15) reduces resistance to 5-FU in CRC cells by competitively binding to miR-31 (107). The decreased expression of lncRNA solute carrier family 25 member 25-antisense RNA 1 (SLC25A25-AS1) promotes the proliferation of CRC cells, resistance to 5-FU and DOX, and EMT (108). Ren et al. showed that knockdown of lncRNA H19 increases the sensitivity of HT-29 cells to MTX, whereas upregulation of lncRNA H19 increases resistance to MTX. These findings indicated that lncRNA H19 is a contributive factor to the development of resistance to MTX (109). LncRNA H19 mediates resistance to MTX in CRC on condition that the Wnt/β-catenin pathway is activated (109). Through the miR-186/CPEB2 axis, lncRNA taurine upregulated 1 (TUG1) affects resistance to MTX in CRC (110). Some studies indicated that lncRNAs are the key factors in the development of resistance to radiation in CRC. For example, lncRNA opa-interacting protein 5-antisense RNA 1 (OIP5-AS1) and dual specificity tyrosine phosphorylation regulated kinase 1A (DYRK1A) are downregulated in radiation-tolerant CRC cell lines (111). Moreover, studies using qRT-PCR detected a meaningful increase in the expression of lncRNA UCA1 in CRC tissues after radio-chemotherapy (112). Downregulation of lncRNA UCA1 enhances the radio-sensitivity of CCL244 cells by blocking colony formation and proliferation, as well as promoting apoptosis through inducement of radiation (112). Yang et al. found that lincRNA-ROR is upregulated in CRC cell lines and tissue samples (44). They further manifested that silencing of lincRNA-ROR increases sensitivity to radiation therapy against CRC by suppressing cell viability and facilitating apoptosis (44). Moreover, in xenotransplantation models, the combination of specific knockout of lincRNA-ROR and radiotherapy can markedly reduce tumor growth (44). Thus far, some evidence suggests that lncRNA is a major factor involved in the development of drug resistance in CRC.

## Discussion and Conclusions

In this review, we introduce the recently reported mechanism of lncRNAs in CRC, highlighting their biological importance and therapeutic applications. CRC is characterized by a high recurrence rate, intense metastatic potential, and low detection rate; it is currently the second and third most common cause of cancer-related death in men and women, respectively (113). As mentioned earlier, an accumulating body of evidence suggests that lncRNAs are vital for the proliferation, invasion, and metastasis of CRC cells. These studies support the notion that lncRNAs are critical therapeutic targets in advanced CRC. lncRNAs are also functional regulators involved in autophagy in CRC. Recently, scientists stated that autophagy may inhibit the occurrence of cancer (114). Therefore, lncRNAs may be an important determinant of CRC. Furthermore, lncRNAs are strongly linked to drug resistance in CRC. Thus, targeting lncRNAs may reverse sensitivity to drugs in this setting. Collectively, the evidence suggests that lncRNA represents a very promising biomarker in patients with CRC. Moreover, the lncRNA-mediated treatment of patients with CRC is also encouraging.

lncRNAs are useful in non-invasive screening. Exosome secretions occur in cells and body fluids (plasma, urine, cerebrospinal fluid, saliva, etc.). Conveniently, there are many sources of exosomes *in vivo*, and exosomes have potential diagnostic value (115). Exosomes are characterized by favorable bioavailability, distribution, and stability *in vivo* and *in vitro*, as well as their ability to cross the blood-brain barrier, and regulate gene expression of target cells by transferring miRNAs and small interfering RNA. These characteristics render them superior to other extracellular vesicles as potential therapies (115). In addition, exosomes contain lncRNAs, and the exosome lncRNA UCA1 can be separated from the blood serum of patients with CRC (98). The circulating exosomes containing lncRNA UCA1 can be used as an evaluation factor for the clinical efficacy of cetuximab in patients with CRC. Notably, patients with progressive disease/stable disease have significantly higher levels of lncRNA UCA1 than those with partial/complete remission (98). Besides, exosomes originated in cetuximab-resistant cells can alter the expression of lncRNA UCA1 and enhance resistance to cetuximab in CRC cells (98). This indicates that lncRNAs have great potential as markers for the effective diagnosis and targeted therapy of CRC.

However, a number of studies have two major limitations. Firstly, most studies focused on cells and tissues. Further animal model investigations, human pathophysiological clinical trials, and basic research studies are required to validate the spectrum of lncRNAs as diagnostic indices and assess the effectiveness of lncRNAs-based therapies in clinical practice. Secondly, the specific mechanism through which lncRNAs impact on CRC remains unclear. It is necessary to optimize the experimental conditions and scientifically explore and analyze the relationship between lncRNAs and CRC with the help of advanced experimental technology.

## Author Contributions

QH, JL, YY, and WZ: conceptualization. QH, JL, YY, YL, XL, and ZL: writing—original draft preparation. All authors: reviewing and editing.

### Conflict of Interest

The authors declare that the research was conducted in the absence of any commercial or financial relationships that could be construed as a potential conflict of interest.
