# Supplementary material for: Emerging Roles of lncRNAs in the Formation and Progression of Colorectal Cancer
Source: Front Oncol. 2020 Jan 17;9:1542. doi: 10.3389/fonc.2019.01542 (PMC6978842; doi:10.3389/fonc.2019.01542)
Supplement: Supplementary file 1 [file Table_1.DOC]

**SUPPLEMENTARY TABLE 1** Expression levels and regulatory pathways of various lncRNAs as ceRNAs in CRC

| **LncRNA** | **Expression level in CRC** | **Signaling pathway** | **Reference** | **Functions** |
| --- | --- | --- | --- | --- |
| **NEAT1** | **Upregulated** | **1.NEAT1/miR-193a-3p / KRAS**  **2.NEAT1/miR-196a-5p/GDNF** | **1.PMID:30575330**  **2.PMID:30383193** | **Promotes cellular proliferation, migration, and suppresses apoptosis** |
| **HAGLROS** | **Upregulated** | **HAGLROS/miR-100/ATG5 axis** | **PMID:30430634** | **Inhibits apoptosis and autophagy** |
| **XIST** | **Upregulated** | **1.XIST/miR-133a-3p/RhoA**  **2.XIST / miR-486-5p/NRP-2**  **3.XIST/miR-124 / SGK1**  **4.XIST/miR-200b-3p/ ZEB1** | **1.PMID:30678736**  **2.PMID:30656681**  **3.PMID:30439718**  **4.PMID:28837144** | **1.and 2.Promotes cytoskeletal reorganization and migration**  **3.Enhances Doxorubicin resistance**  **4.Promotes  cellular proliferation, invasion, EMT and CRC stem cell generation** |
| **SNHG6** | **Upregulated** | **1.SNHG6/miR-181a-5p/E2F5**  **2. SNHG6/miR-26a/b and miR-214/EZH2**  **3.SNHG6/miR-760/FOXC1** | **1.PMID:30666158**  **2.PMID:30626446**  **3.PMID:30254467** | **Promotes cellular proliferation, migration, and suppresses apoptosis** |
| **LINC00483** | **Upregulated** | **LINC00483/miR-204-3p/FMNL2** | **PMID:30594388** | **Promotes cellular proliferation and metastasis** |
| **FAL1** | **Upregulated** | **FAL1/miR-637/NUPR1** | **PMID:30267804** | **Promotes cellular proliferation, migration and invasion** |
| **ZNFX1** | **Upregulated** | **1.ZNFX1-AS1-miR-144-EZH2**  **2.ZNFX1/miR-150-5p/VEGFA** | **1.PMID:30770796**  **2.PMID:30250022** | **Promotes cellular proliferation, migration, angiogenesis** |
| **BRM** | **Upregulated** | **BRM/miR-204-3p/TPT1** | **PMID:30563768** | **Promotes cellular proliferation, migration and invasion** |
| **TP73-AS1** | **1.Downregulated**  **2.Upregulated** | 1.TP73-AS1/miR-103/PTEN **2.TP73-AS1/miR-194/TGFα** | **1.PMID:30472379**  **2.PMID:30010111** | **1.Inhibits CRC cell growth**  **2.Promotes cell proliferation, migration and invasion** |
| **LOC101927746** | **Upregulated** | **LOC101927746/miR-584-3p/SSRP1** | **PMID:30616889** | **Promotes cellular proliferation, migration and invasion** |
| **CACS15** | **Upregulated in OXA-**  **resistant CRC tissues and cells** | **CACS15/miR-145/ABCC1** | **PMID:30639170** | **Contributes to oxaliplatin resistance** |
| **FOXD2-AS1** | **Upregulated** | **FOXD2-AS1/miR‐185‐5p/CDC42** | **PMID:29737580** | **Promotes cellular proliferation, migration and invasion** |
| **OIP5-AS1** | **Downregulated in CRC cell of radio-**  **resistance** | **OIP5-AS1/miR-369-3p/DYRK1A** | **PMID:29773344** | **Suppresses cell viability, increases radio-induced apoptosis capacity, and enhances radio-sensitivity** |
| **GACAT3** | **Upregulated** | **GACAT3/miR-149/SP1 and STAT3** | **PMID:29593420** | **Promotes cellular proliferation, migration and invasion** |
| **HAND2-AS1** | **Downregulated** | **HAND2-AS1/miR-1275/KLF14** | **PMID:30078677** | **Suppresses progression** |
| **TINCR** | **Downregulated** | **TINCR/miR-107/CD36** | **PMID:30521471** | **Suppresses cellular proliferation, migration, and promotes apoptosis** |
| **SCARNA2** | **Upregulated** | **SCARNA2/miR-342-3p-EGFR/BCL2** | **PMID:30443961** | **Promotes chemoresistance** |
| **SNHG5** | **Upregulated** | **SNHG5/miR-132-3p/CREB5** | **PMID:30395767** | **Promotes proliferation, migration and metastasis** |
| **NORAD** | **Upregulated** | **NORAD/miR-202-5p** | **PMID:29471886** | **Promotes cellular proliferation, migration and metastasis** |
| **FTX** | **Upregulated** | **FTX/ miR-215 and inhibits phosphorylation of vimentin** | **PMID:29925853** | **Promotes cellular proliferation, migration and metastasis** |
| **ZEB1-AS1** | **Upregulated** | **ZEB1-AS1/miR-101/ZEB1** | **1.PMID:29511455** | **Promotes cellular proliferation, migration and metastasis** |
| **ZFAS1** | **Upregulated** | **1.ZFAS1/miR-484**  **2.ZFAS1/miR-150-5p/VEGFA** | **1.PMID:29179614**  **2.PMID:30250022** | **1.Promotes cellular proliferation, migration and metastasis**  **2.Suppresses cellular proliferation, migration, invasion and angiogenesis** |
| **HOTAIR** | **Upregulated** | **1.HOTAIR/miR-203a-3p/ Wnt/β-catenin signaling pathway**  **2.HOTAIR/miRNA-545/EGFR** | **1.PMID:29680837**  **2.PMID:28364379** | **1.Promotes CRC progression and increases chemoresistance** |
| **DANCR** | **Upregulated** | **DANCR/miR-577/HSP27** | **PMID:29717105** | **Promotes cellular proliferation, migration and metastasis** |
| **H19** | **Upregulated** | **1.H19/miR-194-5p/SIRT1**  **2.H19/miR-141/β-catenin pathway**  **3.H19/miR-29b-3p/PGRN Axis**  **4.H19/miR-200a/β-catenin**  **5. H19/ miR-138 and miR-200a/ Vimentin, ZEB1, and ZEB2** | **1.PMID:30451820**  **2.PMID:30083271**  **3.PMID:29754471**  **4.PMID:28164117**  **5.PMID:26068968** | **1.Promotes autophagy**  **2.Promotes the stemness and chemoresistance**  **3.Promotes EMT progression**  **4.Promotes cell proliferation**  **5. Promotes EMT progression and tumor growth** |
| **LINC00473** | **Upregulated** | **LINC00473/ miR-15a/?** | **PMID:30126852** | **Promotes the Taxol resistance** |
| **MEG3** | **Downregulated in oxaliplatin-**  **resistant CRC tissues and cell.** | **MEG3/miR-141/PDCD4** | **PMID:30119236** | **Reduce the oxaliplatin resistance** |
| **TUG1** | **1.Upregulated**  **2.Upregulated  in MTX-resistant CRC** | **1.TUG1/miR-600/KIAA1199**  **2.TUG1/miR-186/CPEB2** | **1.PMID:29776371**  **2.PMID:28302487** | **1.Promotes cellular proliferation, migration and metastasis**  **2.Mediates MTX resistance** |
| **LINC00174** | **Upregulated** | **LINC00174/miR-1910-3p/TAZ** | **PMID:29729381** | **Promotes progression** |
| **LINC00675** | **Downregulated** | **LINC00675/miR-942/Wnt/β-catenin signaling** | **PMID:29524886** | **Inhibits cellular proliferation and metastasis** |
| **BANCR** | **Upregulated** | **BANCR/miR-203/CSE1L** | **PMID:30144787** | **Promotes tumorigenesis and enhances adriamycin resistance** |
| G GAPLIN | **Upregulated** | GAPLINC/miR-34a/c-MET signal pathway | **PMID:29427222** | **Promotes cellular migration and invasion** |
| **LINC01503** | **Upregulated** | **LINC01503/miR-4492/FOXK1** | **PMID:30542444** | **Promotes cellular proliferation and invasion** |
| **MIAT** | **Upregulated** | **MIAT/miR-132/Derlin-1** | **PMID:29686537** | **Promotes growth and metastasis** |
| **LINC01296** | **Upregulated** | **LINC01296/miR-26a/GALNT3** | **PMID:30547804** | **Promotes the tumor formation, liver metastasis and cellular chemoresistance.** |
| **HIF1A-AS2** | **Upregulated** | **HIF1A-AS2/miR-129-5p/DNMT3A** | **PMID:29278853** | **Promotes the progression and EMT formation** |
| **SNHG7** | **Upregulated** | SNHG7/miR-216b/GALNT1 | **PMID:29915311** | **Promotes cellular proliferation and liver metastasis** |
| **HOXD-AS1** | **Upregulated** | **HOXD-AS1/miR‑217** | **PMID:29749477** | **Promotes cellular proliferation, invasion, EMT and stem cell formation** |
| **ENST00000547547** | **Downregulated in 5-FU-resistant CRC cells** | **ENST00000547547/miR-31** | **PMID:29115526** | **Suppresses 5-fluorouracil resistance** |
| **LINC00312** | **Downregulated** | LINC00312/miR-21/PTEN | **PMID:30134003** | **Suppresses cellular proliferation and metastasis** |
| **OECC** | **Upregulated** | **OECC/miR-143-3p/NF-κB and p38 MAPK pathways.** | **PMID:30126634** | **Promotes cellular proliferation, migration, and suppresses apoptosis** |
| **ABHD11-AS1** | **Upregulated** | **ABHD11-AS1/miR-1254/WNT11** | **PMID:30537177** | **Promotes cellular proliferation, migration** |
| **MAFG-AS1** | **Upregulated** | **MAFG-AS1/miR-147b/NDUFA4** | **PMID:30348529** | **Promotes cellular proliferation, cell cycle progression, and invasion, and inhibits apoptosis** |
| **HEIH** | **Upregulated** | **HEIH/miR-939/Bcl-xL** | **PMID:29081216** | **Promotes cellular proliferation and decreases apoptosis** |
| **GAS5** | **Downregulated** | **GAS5/miR‑182‑5p/FOXO3a** | **PMID:30066886** | **Inhibits cellular proliferation and promotes apoptosis** |
| **LINC00152** | **1.Downregulated**  **2.Upregulated** | **1.LINC00152/miR-376c-3p/Ki-67, Bcl-2, and Fas.**  **2.LINC00152/miRNA‑206/NRP1** | **1.PMID:28078002**  **2.PMID:29956750** | **1. Decreases cellular viability and increased apoptosis**  **2. Enhances cellular proliferative and invasive ability** |
| **CASC7** | **Downregulated** | **CASC7/miR-21/ING3** | **PMID:28954383** | **Inhibits proliferation and migration** |
| **SOX21-AS1** | **Upregulated** | **SOX21-AS1/miR-145/MYO6** | **PMID:29217166** | **Promotes cellular proliferation, invasion** |
| **FBXL19-AS1** | **Upregulated** | **FBXL19-AS1/miR-203/?** | **PMID:28479250** | **Promotes cellular proliferation, migration and metastasis** |
| **SNHG3** | **Upregulated** | **SNHG3/miR-182-5p/c-Myc** | **PMID:28731158** | **Promotes cellular proliferation** |
| **UCC** | **Upregulated** | **UCC/miR-143/？** | **PMID:28492554** | **Promotes cell growth and invasion** |
| **CRNDE** | **Upregulated** | **CRNDE/miR-181a-5p/Wnt/β-catenin signaling** | **PMID:28086904** | **Promotes cellular proliferation and increase chemoresistance** |
| **UICLM** | **Upregulated** | **UICLM/miRNA-215/ZEB2** | **PMID:29187907** | **Promotes cellular EMT, proliferation, invasion, CRC stem cell generation, tumor development and liver metastasis** |
| **ucoo2kmd** | **Upregulated** | **ucoo2kmd/miR-211-3p/CD44** | **PMID:26974151** | **Promotes** **cellular proliferation** |
| **CASC2** | **Downregulated** | **CASC2/miR-18a/PIAS3** | **PMID:27198161** | **Suppresses cellular proliferation and tumor development** |
| **UCA1** | **Upregulated** | **UCA1/miR-204-5p/CREB1/BCL2/RAB22A** | **PMID:27046651** | **Promotes the 5-FU-resistance in CRC cell** |
