# Supplementary material for: Emerging Roles of lncRNAs in the Formation and Progression of Colorectal Cancer
Source: Front Oncol. 2020 Jan 17;9:1542. doi: 10.3389/fonc.2019.01542 (PMC6978842; doi:10.3389/fonc.2019.01542)
Supplement: Supplementary file 2 [file Table_2.DOC]

**SUPPLEMENTARY TABLE 2** Summaries of various lncRNAs as tumor suppressor and tumor promotor in CRC

| **LncRNAs as tumor promotor** | **Reference** |
| --- | --- |
| **NEAT1** | **PMID:30575330**  **PMID :30407674** |
| **LUCAT1** | **PMID:30690837** |
| **HAGLROS** | **PMID:30430634** |
| **MLK7-AS1** | **PMID:30535460** |
| **XIST** | **PMID:30678736**  **PMID:30656681** |
| **SNHG6** | **PMID :30666158**  **PMID :30626446**  **PMID : 30662328**  **PMID : 30254467** |
| **LINC00483** | **PMID : 30594388** |
| **LncTCF7** | **PMID : 30225781**  **PMID : 29532890**  **PMID : 29344178** |
| **FAL1** | **PMID : 30267804**  **PMID : 30290064** |
| **ZNFX1-AS1** | **PMID : 30770796** |
| **RUNX1-IT1** | **PMID : 30499136** |
| **CCAT1** | **PMID : 30615124** |
| **lncBRM** | **PMID : 30563768** |
| **TP73-AS1** | **PMID : 30472379**  **PMID : 30010111** |
| **MAPKAPK5-AS1** | **PMID :  30343528** |
| **SNHG15** | **PMID : 30317592** |
| **Hotair** | **PMID : 30362162**  **PMID : 29808247** |
| **LOC101927746** | **PMID : 30616889** |
| **PVT1-214** | **PMID : 30076414** |
| **TPTE2P1** | **PMID : 30382596** |
| **FOXD2-AS1** | **PMID : 29737580**  **PMID : 28925486** |
| **LINC00365** | **PMID : 30525245** |
| **GACAT3** | **PMID : 29593420** |
| **LINC01510** | **PMID : 30536314** |
| **SNHG1** | **PMID : 29509245**  **PMID : 29340086** |
| **uc.338** | **PMID : 29901203** |
| **EWSAT1** | **PMID : 30402843** |
| **SNHG5** | **PMID : 30395767** |
| **NORAD** | **PMID : 29471886** |
| **u50535** | **PMID : 29970882** |
| **SLCO4A1-AS1** | **PMID : 30201010** |
| **FTX** | **PMID : 29925853** |
| **CRNDE** | **PMID : 30334449**  **PMID : 28594403** |
| **AB073614** | **PMID : 29439310** |
| **ZEB1-AS1** | **PMID : 29511455** |
| **ZFAS1** | **PMID : 29179614**  **PMID : 30250022** |
| **ATB** | **PMID : 29692736** |
| **DANCR** | **PMID : 29717105** |
| **CYTOR** | **PMID : 30064438** |
| **NORAD** | **PMID : 30349308** |
| **PVT1** | **PMID : 29512788** |
| **LINC00659** | **PMID : 29523145** |
| **TUG1** | **PMID : 29776371**  **PMID : 27421138**  **PMID : 26856330** |
| **PlncRNA-1** | **PMID : 28835319** |
| **LINC00174** | **PMID : 29729381** |
| **RP11-362K14.5 (CCSlnc362)** | **PMID : 30518759** |
| **SNHG7** | **PMID : 29915311** |
| **HOTTIP** | **PMID : 30229808** |
| **VIM-AS1** | **PMID : 29656793** |
| **SH3PXD2A-AS1** | **PMID : 29734178** |
| **RP1-85F18.6** | **PMID : 30226619** |
| **BANCR** | **PMID : 30144787**  **PMID : 25013510** |
| **ZEB1-AS1** | **PMID : 29886791** |
| **GAPLINC** | **PMID : 29427222** |
| **RP11-317J10.2** | **PMID : 29073791** |
| **LINC01503** | **PMID : 30542444** |
| **FAM83H-AS1** | **PMID : 29434883** |
| **MIAT** | **PMID : 29686537** |
| **DLEU7-AS1** | **PMID : 29364477** |
| **HOTTIP** | **PMID : 29274585** |
| **DLEU1** | **PMID : 30098595** |
| **LINC01296** | **PMID : 30547804** |
| **HIF1A-AS2** | **PMID : 29278853** |
| **LINC00460** | **PMID : 30092404** |
| **XLOC_010588** | **PMID : 29436686** |
| **SNHG7** | **PMID : 29970122** |
| **HOXDAS1** | **PMID : 29749477** |
| **BCYRN1** | **PMID : 30114690** |
| **CCHE1** | **PMID : 30484108** |
| **H19** | **PMID : 29754471**  **PMID : 28164117** |
| **MAFG-AS1** | **PMID : 30348529** |
| **HEIH** | **PMID : 29081216** |
| **Lnc00152** | **PMID : 29956750** |
| **LINC01510** | **PMID : 29581707** |
| **HNF1A-AS1** | **PMID : 29145164** |
| **ANCR** | **PMID : 27983539** |
| **SOX21-AS1** | **PMID : 29217166** |
| **SNHG12** | **PMID : 28225893** |
| **TUC.338** | **PMID : 27914101** |
| **CHRF** | **PMID : 28430582** |
| **RP11-708H21.4** | **PMID : 28427191** |
| **BLACAT1** | **PMID : 28277544** |
| **SBDSP1** | **PMID : 27890432** |
| **SPRY4-IT1** | **PMID : 27391336** |
| **FBXL19-AS1** | **PMID : 28479250** |
| **PCAT-1** | **PMID : 28855110** |
| **SNHG17** | **PMID : 28933484** |
| **PANDAR** | **PMID : 27629879** |
| **BC032913** | **PMID : 28918047** |
| **DUXAP10** | **PMID : 28779166** |
| **PURPL** | **PMID : 28877474** |
| **FOXP4-AS1** | **PMID : 27790757** |
| **ROR** | **PMID : 28216611** |
| **SNHG3** | **PMID : 28731158** |
| **LINC-UBC1** | **PMID : 28260919** |
| **UICLM** | **PMID : 29187907** |
| **GHET1** | **PMID : 27931286** |
| **CASC11** | **PMID : 27012187** |
| **UFC1** | **PMID : 27195675** |
| **HULC** | **PMID : 27496341** |
| **GAPLINC** | **PMID : 27259250** |
| **PRNCR1** | **PMID : 26530130** |
| **uc002kmd.1** | **PMID : 26974151** |
| **AFAP1-AS1** | **PMID : 27261589**  **PMID : 27578191** |
| **ZFAS1** | **PMID : 26506418** |
| **ANRIL** | **PMID : 27314206** |
| **DQ786243** | **PMID : 26934980** |
| **POU3F3** | **PMID : 26510906** |
| **CCAL** | **PMID : 25994219** |
| **Sox2ot** | **PMID : 27353770** |
| **HOXA** | **PMID : 26678886** |
| **MALAT1** | **PMID : 26887056**  **PMID : 25446987** |
| **FEZF1-AS1** | **PMID : 26848625** |
| **SPRY4-IT1** | **PMID : 27621655** |
| **DANCR** | **PMID : 26617879** |
| **ATB** | **PMID : 25750289** |
| **LOC554202** | **PMID : 26362196** |
| **91H** | **PMID : 25058480** |
| **UCA1** | **PMID : 30362538**  **PMID : 26238511** |
| **HOXA-AS2** | **PMID : 29118916**  **PMID: 28112720** |
| **LnRNAs as tumor suppressor** | **Reference** |
| **GAS5** | **PMID : 30672001**  **PMID : 29308053**  **PMID : 30066886**  **PMID : 28521420** |
| **ST3Gal6-AS1** | **PMID : 30613961** |
| **ENST00000547547** | **PMID : 30542703** |
| **OIP5-AS1** | **PMID : 29773344** |
| **MEG3** | **PMID : 29628342** |
| **HAND2-AS1** | **PMID : 30078677** |
| **kcna3** | **PMID : 30099342** |
| **BC0209135** | **PMID : 29949151** |
| **TINCR** | **PMID : 30521471**  **PMID : 28418933** |
| **CPS1-IT1** | **PMID : 29017924** |
| **LINC00460** | **PMID : 30123352** |
| **lncRNA-422(ENST00000415820)** | **PMID : 29050940** |
| **LINC00675** | **PMID : 29524886** |
| **LINC00312** | **PMID : 30134003** |
| **OCC-1** | **PMID : 29931370** |
| **TMEM75** | **PMID : 29964097** |
| **OECC** | **PMID : 30126634** |
| **ABHD11-AS1** | **PMID : 30537177** |
| **CRCMSL** | **PMID : 30575817** |
| **DILC** | **PMID : 29621586** |
| **HNF1AAS1** | **PMID : 28791380** |
| **CASC7** | **PMID : 28954383** |
| **TUSC7** | **PMID : 28214867** |
| **CPS1-IT1** | **PMID : 29145177** |
| **BCAT1** | **PMID : 28416735** |
| **AB073614** | **PMID : 28738539** |
| **NNT-AS1** | **PMID : 27966450** |
| **LINC00959** | **PMID : 29228592** |
| **HOXB-AS3** | **PMID : 28985503** |
| **UCC** | **PMID : 28492554** |
| **NONHSAT062994** | **PMID : 28978149** |
| **LINC00152** | **PMID : 28078002** |
| **CTD903** | **PMID : 27035092** |
| **GNAT1-1** | **PMID : 27912775** |
| **HOTAIRM1** | **PMID : 27307307** |
| **SLC25A25-AS1** | **PMID : 27553025** |
| **LINC01133** | **PMID : 27443606** |
| **CASC2** | **PMID : 27198161** |
